# Genotypic and phenotypic heterogeneity in *Streptococcus mutans* isolated from diabetic patients in Rome, Italy

**DOI:** 10.1186/s40064-016-3470-0

**Published:** 2016-10-13

**Authors:** Arpan De, Guido Pasquantonio, Loredana Cerroni, Dezemona Petrelli, Davide Lauro, Marta Longhi, Luca A. Vitali

**Affiliations:** 1 Microbiology Unit, School of Pharmacy, University of Camerino, Via Gentile III da Varano, 62032 Camerino, MC Italy; 2 Department of Clinical Science and Translational Medicine, University of Rome Tor Vergata, Rome, Italy; 3 School of Biosciences and Veterinary Medicine, University of Camerino, Camerino, Italy; 4 Department of Systems Medicine, University of Rome Tor Vergata, Rome, Italy

**Keywords:** Caries, Plaque, PFGE, Antimicrobial susceptibility

## Abstract

Our study focuses on the antimicrobial susceptibility, genotypic and phenotypic heterogeneity, and serotype classification of the *Streptococcus mutans* isolated from type II diabetic patients (n = 25; age 42–68). Eighty-two percent of isolates were classified as serotype *c*. No serotype *k* was present. Macrorestriction analysis of genomic DNA of the isolates exhibited a clonal diversity that paralleled the phenotypic heterogeneity, which was also assessed in terms of biofilm forming ability. Isolates were susceptible to all the classes of antibiotics. In conclusion a great heterogeneity and no antimicrobial resistance were apparent in the considered *S. mutans* strains from diabetic patients.

## Background

Globally, dental caries is a major public health concern imposing an economic impact among developed countries. Italy was estimated with the highest percentage of Gross National Product spent on oral health in 2010 (Patel Reena 2012). Predisposition of diabetic patients to dental caries may result as sequelae of poor glycemic control, due to high levels of glucose in saliva in conjunction with xerostomia, although a strong association has not been found hitherto (King et al. 1998; Ship 2003; Taylor 2004). *Streptococcus mutans*, one of the main causative agents of caries (Loesche 1986), is a commensal inhabitant of the tooth surface, participating in the formation of a complex multispecies microbial community (i.e. a biofilm) called dental plaque (Kuramitsu et al. 2007). It adheres to the pellicle of tooth enamel surface through adhesins (AgI/II) or extracellular polysaccharide (EPS) formed by glucosyltransferases (GtfB, GtfC, and GtfD) in presence of sucrose (Krzyściak et al. 2014). Increased intake of dietary carbohydrates acts as source of sugar increasing the production of metabolic acid end-products, and thus demineralization of teeth enamel and dentine at low pH (Loesche 1986). The EPS also serves as a reservoir of sugar and maintains acidic pH in the depths of plaque (Dibdin and Shellis 1988), and a continuous flush of salivary sugar in the EPS may pose a greater caries threat in diabetics. In a biofilm, *S. mutans* expresses competence and bacteriocin genes that can facilitate integration of various dispensable genes (*cnm, gbpA*), endowing a competitive advantage and a wide array of genome heterogeneity (Waterhouse and Russell 2006; Waterhouse et al. 2007). In general, Horizontal Gene Transfer (HGT) is known to be strongly favored in biofilm increasing the acquisition rate of exogenous genetic material. Among this material are the determinants of antibiotic resistance (Davies and Davies 2010), posing a continuous threat to the antimicrobial susceptibility of *S. mutans* (Baddour et al. 2005). In this context, diabetic individuals are subjected to various long term medications escalating the chances of acquiring resistance to these medicines by the indigenous flora of diabetics. Frequent use of antibiotics against infections in such immune-compromised patients has shown increased risk of antibiotic resistance among pathogens (Boyanova and Mitov 2013).

Based on the rhamnose glucose polysaccharide surface antigen (RGP) *S. mutans* is classified into four serotypes (*c*, *e*, *f*, *k*), among which *k* exhibits reduced cariogenecity and immunogenicity (Hamada and Slade 1980; Nakano et al. 2010). In addition, infective endocarditis (IE) is reportedly caused at higher frequency by non-*c* serotype *S. mutans* (Nakano et al. 2010). Also DNA fingerprinting methods such as RFLP, AP-PCR, PFGE have demonstrated significant genotypic diversity among *S. mutans* isolates (Caufield and Walker 1989; Brady et al. 1991; Napimoga 2004; Lembo et al. 2007; Moser et al. 2010).

In the present study, we have focused on the antimicrobial susceptibility as well as genotypic and phenotypic heterogeneity of *S. mutans* isolates from type II diabetic patients aged >40 and bring the importance of such studies from patients with metabolic disorders to the forefront.

## Results and discussion

### Dental health analysis of diabetes

The DMF index is a key measure of the caries experience in dental epidemiology. The mean DMFT values among the Italian middle aged diabetic patients was 16.3 (SD = 9.0) while the median plaque index was 0.544. Previous oral health studies among Type II diabetic population in Sudan also found a significant higher plaque index (Mohamed et al. 2013). Moreover, Taylor et al. (2004) found six large cohort studies reporting an association of poor glycemic control with high caries experience. Diabetic subjects are usually restricted to refined carbohydrates and proteinaceous diet. Nevertheless, poor glycemic control, xerostomia and repeated food intake may disrupt the microbial homeostasis of plaque and increase the incidence of caries due to acidogenic bacteria (Taylor 2004; Marsh 2009).

### Screening of *S. mutans* isolates and serotyping

500 bp amplicons obtained using *gtf*B primers confirmed the putative *S. mutans* clinical isolates from diabetics. Only 68 % (n = 17) isolates from the serially diluted plaque or saliva samples showed positive amplification. Selection of adult patients in a particular age group with no other medical complications except diabetes might have restricted to end up with low number of patients and consequently isolating few clinical strains. Even though possessing a higher plaque index, the viable count of *S. mutans* was low presumably due to proper maintenance of their oral health by 2 times brushing and usage of mouth washes among 48 % respondents (data not shown). Screening of the serotype by a molecular approach classified 14 isolates as *c* type, while 2 and 1 as type *e* and *f*, respectively. We did not record any *k* serotype among our isolates. This result is in accordance with the global prevalence of serotype *c S. mutans* in the oral cavity (Nakano et al. 2006). Serotype *e*, *f*, *k* are commonly found in atheromatous plaque (Abranches et al. 2011). People with diabetes are usually more prone to heart disease and atherosclerosis (Chait and Bornfeldt 2009), which may follow dental surgical procedures favoring the access of *S. mutans* in sufficient numbers in the blood-stream. Hence, a thorough stomatological study of the diabetic subjects can serve as a prophylaxis to such health disorder.

### Antimicrobial susceptibility testing

Diabetic foot infection, urinary tract and lower respiratory tract infections are a constant threat to poor glycemic control, which has demonstrated an increase in antibiotic prescription rates in Netherlands (Venmans et al. 2009). Notwithstanding this fact prompts a warning situation, pointing towards increased antimicrobial resistance among indigenous flora, all the bacterial strains isolated from diabetics were susceptible to all antibiotics tested (Table 1). According to EUCAST clinical breakpoint, the average zone diameter against penicillin, clindamycin and vancomycin were far from the breakpoints (12, <19 and 15 mm, respectively). Concomitantly, the susceptibility against other antibiotics (erythromycin, oxacillin, rifampicin, tetracycline, gentamicin, cefoxitin, linezolid, norfloxacin and levofloxacin) was compared with the performance standard values published by CLSI for streptococci other than *S. pneumoniae* (CLSI 2011). Clindamycin, erythromycin, rifampicin and cefoxitin exhibited higher inhibition compared to others with a zone diameter ranging between 35–41, 30–38, 29–36 and 28–34 mm respectively. Penicillin resistance was not observed, which countertrends the previously recorded 14 % penicillin-resistant *S. mutans* isolates from dental patients in Rome (Pasquantonio et al. 2012). Hence, with scarce available information on antimicrobial disc susceptibility tests based on EUCAST and CLSI guidelines, our work may aid as a reference scale for determining reduced susceptibility to various class of antibiotics for future studies.

**Table 1:** Distribution of *S. mutans* isolates from diabetic patients according to the inhibition zone diameters (mm) exhibited by various antibiotics (EUCAST format)

Antibiotic	Disk Content (μg)	Inhibition zone diameter (mm)																														
		12	13	14	15	16	17	18	19	20	21	22	23	24	25	26	27	28	29	30	31	32	33	34	35	36	37	38	39	40	41	42
Vancomycin	5	0	0	0	0	0	1	6	5	1	4	1*	0	0	0	0	0	0	0	0	0	0	0	0	0	0	0	0	0	0	0	0
Erythromycin	30	0	0	0	0	0	0	0	0	0	0	0	0	0	0	0	0	0	0	1	2	2	1	5	2	2	1	2*	0	0	0	0
Oxacillin	1	0	0	0	0	0	0	0	0	0	0	0	0	3	3	2	5*	3	2	0	0	0	0	0	0	0	0	0	0	0	0	0
Penicillin	1	0	0	0	0	0	0	0	0	0	0	0	0	0	0	0	1	2	3	4	4*	2	1	1	0	0	0	0	0	0	0	0
Rifampicin	5	0	0	0	0	0	0	0	0	0	0	0	0	0	0	0	0	0	3	2	3	3	0	3	3*	1	0	0	0	0	0	0
Tetracycline	30	0	0	0	0	0	0	0	0	0	0	0	0	1	1	0	1	4	4	1	2	2*	1	1	0	0	0	0	0	0	0	0
Gentamicin	10	0	0	0	2	1	2	3	1	3	4	0	0	2*	0	0	0	0	0	0	0	0	0	0	0	0	0	0	0	0	0	0
Cefoxitin	30	0	0	0	0	0	0	0	0	0	0	0	0	0	0	0	0	1	2	2	0	6	4	3*	0	0	0	0	0	0	0	0
Linezolid	10	0	0	0	0	0	0	0	0	0	2	0	1	1	0	4	5*	2	1	2	0	0	0	0	0	0	0	0	0	0	0	0
Norfloxacin	10	1	1	3	1	1	3	2	3	2	1*	0	0	0	0	0	0	0	0	0	0	0	0	0	0	0	0	0	0	0	0	0
Clindamycin	10	0	0	0	0	0	0	0	0	0	0	0	0	0	0	0	0	0	0	0	0	0	0	0	2	1	6	5	0	0	3*	1
Levofloxacin	5	0	0	0	0	0	0	0	1	1	0	4	4	2	3	2*	1	0	0	0	0	0	0	0	0	0	0	0	0	0	0	0

* Zone diameter exhibited by the control strain UA159

### Macrorestriction analysis

**Fig. 1:**
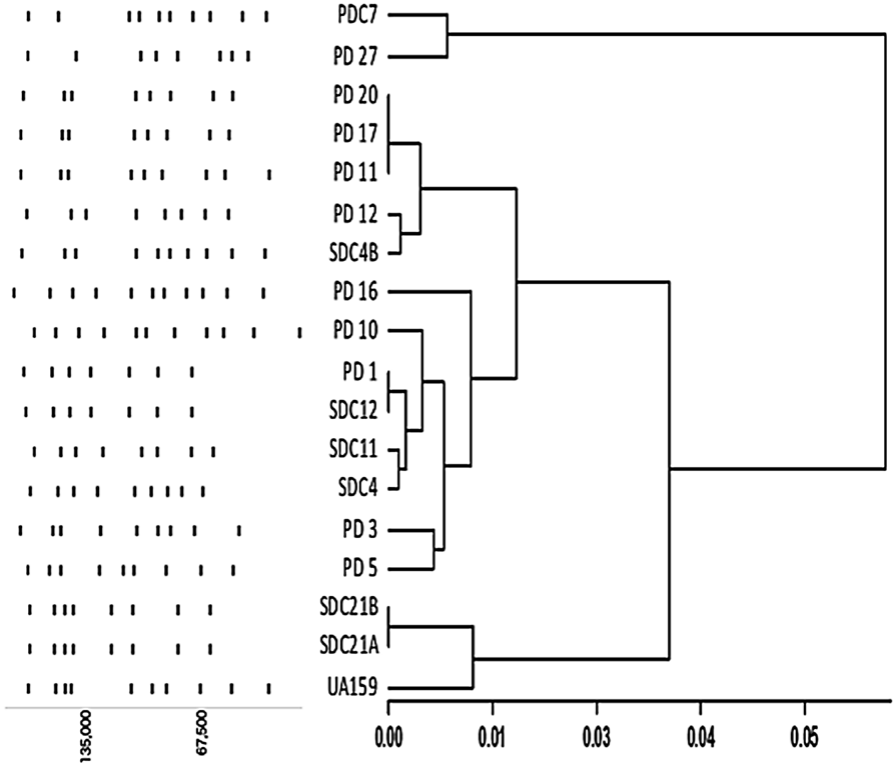
Dendogram and schematic representation of PFGE pattern. Scale represents Pearson distance

Macrorestriction analyses using SmaI and BssHII of *S. mutans* isolates were studied previously to identify maternal transmission as well as genotypic uniformity in an individual (Mineyama et al. 2007; Mitchell et al. 2011). This work illustrates genotypic heterogeneity of *S. mutans* from caries of diabetic patients of a specific region. The two clustered dendogram along with the schematic representation of the digested bands in Fig. 1 illustrates the clonal diversity of the *S. mutans* clinical strains. One major cluster accounts for 88 % of the clinical strains along with reference strain UA159 in it. PD27 and PDC7 formed the other cluster. PFGE of PD11, PD17 and PD20 projected similarity, but PD17 and PD20 showed one less band in the lower part of the gel. Conversely, SDC12 and PD1 exhibited complete overlapping of the digested bands. SDC21A–SDC21B and SDC4–SDC4B were isolated from the oral cavity of the same patient and were included in the study due to their differences in growth behavior in broth. As SDC21A and SDC21B belonged to the same serotype and showed the same macrorestriction pattern, they may be considered as variants of the same strain. SDC4 and SDC4B were instead dissimilar in both the characteristics and may be, therefore, considered as different strains. Genotypic variability is well known in *S. mutans* and records suggests 23 % gene content divergence through whole genome sequence comparison, which validates the diversity found in our study (Cornejo et al. 2013). Although PFGE is considered as the gold standard for discerning clonal relatedness (Birren and Lai 2012), a robust conclusion about the phylogenetic relationship between the strains can be reached only through Whole Genome Sequencing (WGS) studies. WGS of our set of clinical strains are underway, among which the genome sequence of SDC21A has been deposited in GenBank as *S. mutans* AD01 (Accession number: LGAC00000000).

### Phenotypic features of isolates

**Fig. 2:**
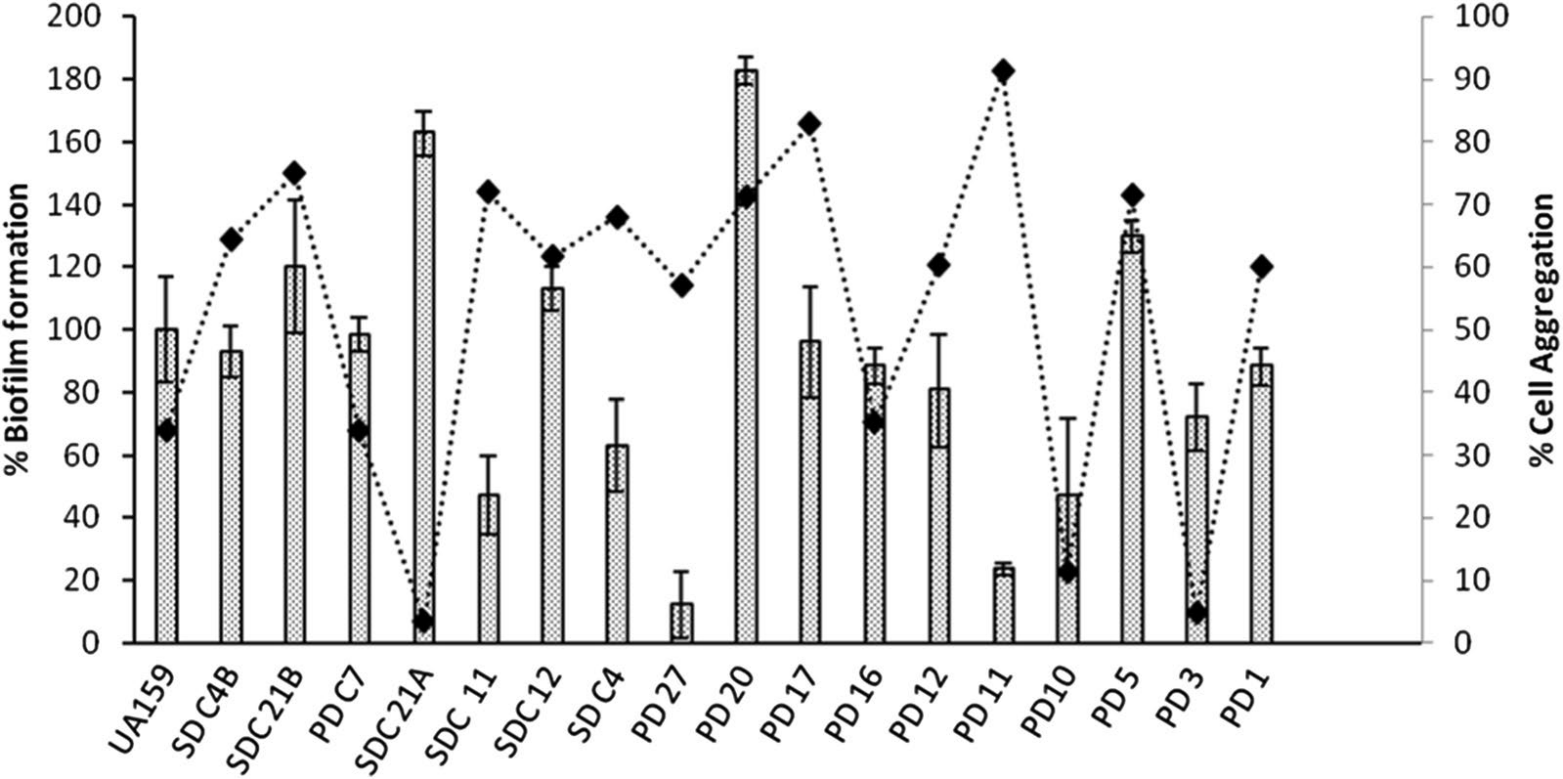
Percentage of biofilm formation (*bars*) and cell aggregation (*squares* and *dotted lines*) among isolates. *Error bar* represents %Relative Standard deviation (RSD)

Biofilm formation, aciduricity and acidogenesis are the key virulence factors which have been extensively compared among large number of clinical isolates with genotypic differences, demonstrating phenotypic variability at the same time (Palmer et al. 2013). Wide genetic variability as observed by PFGE urged us to compare few virulence characteristics among isolates. The strains showed high variability in the capacity to develop mature biofilm. While SDC21A, SDC21B, PD20 and PD5 exhibited high amount of biomass, PD27 and PD11 were poor biofilm formers in presence of glucose Fig. 2. A sucrose independent biofilm formation is governed by various surface antigens among which WapA, P1 and competence factors are known to aid in initial adherence of *S. mutans* to the tooth surface, and a potential variability in these genes among the strains may be associated with such phenotypic heterogeneity (Bowen et al. 1991; Zhu et al. 2006; Senadheera and Cvitkovitch 2008). Gene sequence comparative studies of these gene loci upon WGS may reveal the underlying cause for such phenotypic variation.

Co-aggregation is an important event in the initial stages of biofilm development (Kolenbrander 2000). Apparently there was merely any correlation between amount of biofilm biomass and aggregation (Pearson Correlation r = −0.1), as exemplified by SDC21A and SDC21B, which were high biofilm formers but had a contrasting low aggregation capability Fig. 2. PD27 and PD11 showed an opposite behavior. Hence, co-aggregation is not a major factor determining biofilm formation in *S. mutans*.

Furthermore, the strains were equally acidogenic with a mean final pH of 4.4 ± 0.08 after 72 h (pH range of 4.3–4.6). This may reflect the acidogenic potential of the caries causing bacteria in various patients as well as significant criteria in the multifactorial nature of dental caries. With a mean pH far below the pH of saliva, these bacteria in co-operation with other acidogenic bacteria may pose a serious threat.

## Conclusion

Numerous studies have been conducted on etiology of dental caries in children and young adults (Lembo et al. 2007; Sgolastra et al. 2010). But with an increasing average age of individuals and a globally ascending level of diabetes, it is very essential to make cohort studies specifically focused on oral epidemiology of adult diabetics. Future studies will aim at WGS and comparative sequence analysis of the virulence genes of the clinical strains. Our study also encourages and acts as a reference for future epidemiological studies of *S. mutans* isolates from Italian diabetic patients.

## Methods

### Sampling and growth conditions

Patients were enrolled during the period October 2012–March 2013. Ethical approval was granted by Comitato Etico Indipendente Fondazione PTV Policlinico Tor Vergata (prot. n. 101/12). All the diabetic patients were ≥40 years of age, had ≥10 natural teeth and had no history of long-term antibiotic use (≥14 days) in the 6 months preceding the study. The considered diabetic hosts (n = 25) where diagnosed with a Type II diabetes and had an average age of 58 (SD = 7.2 years). All patients older than 70 (n = 6) were excluded from the study due to various associated combordities. The tooth examination was performed by the same clinician and the dichotomous plaque index (PI) was assessed at four sites per tooth (Löe 1967). For the evaluation of caries experience, the DMFT index (number of decayed, missing and filled teeth) was used. Supragingival plaque on buccal and lingual surface was collected in 10 mL of distilled water. About 5 mL of unstimulated saliva was obtained from all subjects 2 h after the breakfast and 10 min after mouthwash with 10 mL of deionized water. The plaque and saliva samples were homogenized by ultrasonic dispersion for 20 s at 0 °C. 100 µL of these dispersed samples were spread plate on selective agar medium TSY20B (Trypticase Soy agar with 1 % yeast extract, 20 % sucrose and 200 IE of bacitracin) or MSB (Mitis-Salivarius agar containing 15 % sucrose, 1 % tellurite and 200 IE bacitracin) at appropriate dilutions (Gold et al. 1973; Schaeken et al. 1986). All plates were incubated at 37 °C, in an atmosphere of 91 % N_2_, 5 % CO_2_, and 4 % H_2_ for 5 days.

### PCR screening and serotyping

Colonies obtained on selective media were transferred on BHI agar and subsequently screened by colony PCR using *S. mutans* specific primers for *gtf*B (Oho et al. 2000). Similarly, PCR based serotypic classification of the clinical isolates were determined using rhamnose–glucose polysaccharide specific primers (Shibata et al. 2003; Nakano et al. 2004). *S. mutans* UA159 was always used as a positive or negative control as appropriate.

### Susceptibility tests

The antimicrobial susceptibility of the clinical isolates was determined by disc diffusion method based on the protocol prescribed by European Committee on Antimicrobial Susceptibility Testing (EUCAST) on viridans group streptococci (Matuschek et al. 2014). Filter discs (Oxoid Ltd., Hants, United Kingdom and Liofilchem, Italy) containing the antimicrobial agents penicillin (1 U), rifampicin (5 µg), oxacillin (1 µg), clindamycin (10 µg), cefoxitin (30 µg), erythromycin (30 µg), levofloxacin (5 µg), linezolid (10 µg), gentamicin (10 µg), norfloxacin (10 µg), tetracycline (30 µg) and vancomycin (5 µg) were used for the study. The zone diameters were measured at three positions for each disc using a caliper. The antimicrobial susceptibility of *S. mutans* UA159 was evaluated as a reference strain.

### Macrorestriction and PFGE

Genomic DNA extraction from clinical isolates was performed by modification of a protocol described previously (Ripa et al. 2001). A high resolution low background gel picture was used to analyze the DNA fingerprint of clinical isolates. PFGE patterns were analyzed and compared using Gel Analyzer. The molecular weight and retention (Rf) values of the digested bands were calculated based on the molecular weight of λ phage ladder. The band size values of the isolates were compared to create a similarity matrix using Pearson correlation coefficient and subsequently cluster analysis using UPGMA in Infostat Professional version 2014 (Di Rienzo et al. 2014).

### Cell aggregation assay

Clinical isolates were grown in BHI broth till the mid exponential phase (OD_600_ nm = 0.7) from an overnight culture, harvested, washed twice in PBS and re-suspended in the same buffer to obtain an OD_600_ = 0.6 U. One mL of suspension was added with 5 µL of 0.1 M CaCl_2_, vortexed and transferred to a cuvette. After equilibrating at room temperature for 1 min, the decrease in OD_600_ of the samples was recorded for 120 min in a spectrophotometer (Cary 100) at 37° C. The percent of aggregation was calculated as [(OD_600_ at 0 min–OD_600_ at 120 min)/(OD_600_ at 0 min)] × 100 (Ahn et al. 2008).

### Biofilm Assay

Biofilm formation was assessed in polystyrene 96-well (flat bottom) cell culture clusters (Costar 3595; Corning Inc., NY). An overnight culture of each isolate was transferred in pre-warmed BHI and grown at 37 °C in microaerophilic condition till the mid-exponential phase and then diluted 100 fold in Semi Defined Minimal medium containing 0.8 % glucose (Ahn et al. 2008). An aliquot of culture was dispensed in a microtiter plate and incubated at 37 °C, in 5 % CO_2_. After 20 h incubation, the culture medium was decanted and the wells were washed thrice with saline. The adhered cells were stained for 15 min using 200 µL of 0.1 % crystal violet at room temperature. Wells were then rinsed twice with saline (0.9 % NaCl). The bound dye was extracted from the adherent cells using 200 µL of 99 % ethanol and quantified at 495 nm. The assay was performed in triplicates.

### Final pH analysis

Final pH analysis measures pH at which the growth of each isolate is completely inhibited (van Houte et al. 1996). 50 µL of an overnight culture was inoculated in 5 mL of Phenol Red Dextrose broth (Difco) supplemented with 1 % glucose and incubated at 37 °C, 10 % CO_2_ for 3 days. The final pH was determined using a pH meter. Non-inoculated medium was used as a control. The tests were done in triplicates.

### Statistical analysis

Statistical data analysis was done in Statgraphics Centurion ver. XIV and Infostat Professional version 2014 (Di Rienzo et al. 2014). All the data set were analyzed by non parametric Kruskal–Wallis one way ANOVA at 95 % confidence interval.
